# Supplementary material for: A Single-Cell Atlas of Lymphocyte Adaptive Immune Repertoires and Transcriptomes Reveals Age-Related Differences in Convalescent COVID-19 Patients
Source: Front Immunol. 2021 Jul 12;12:701085. doi: 10.3389/fimmu.2021.701085 (PMC8312723; doi:10.3389/fimmu.2021.701085)
Supplement: Supplementary file 1 [file DataSheet_1.pdf]

Supplemental Information

Supplemental Figures 1-9

Supplemental Tables 1-5

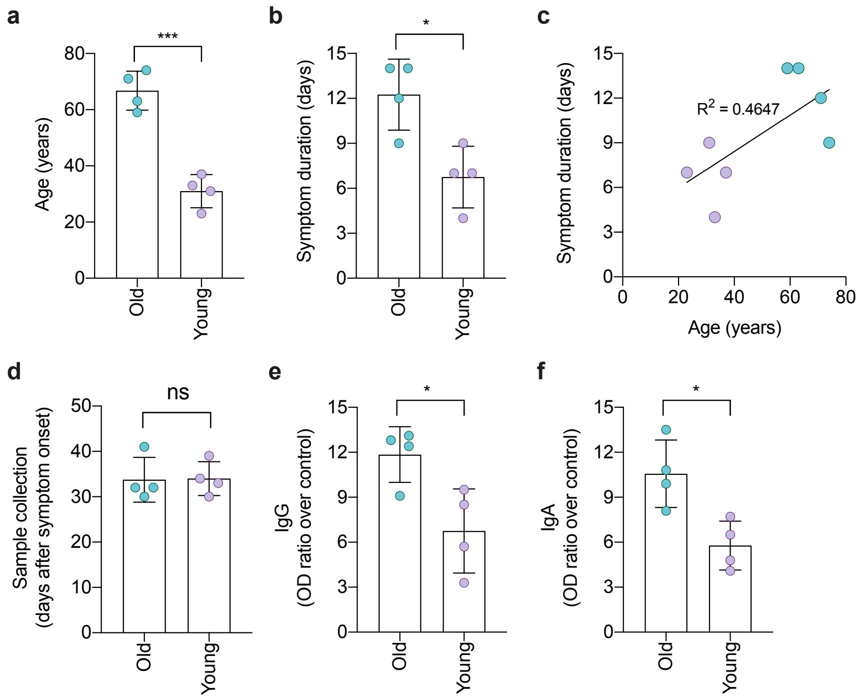

Supplemental Figure 1. **Clinical and serological characteristics of convalescent COVID-19 patients**. **a-b**, Age and COVID-19 symptom duration in patients from the old and young groups. **c**, Linear regression analysis of age and symptom duration in convalescent COVID-19 patients. **d-f**, Serological analysis of convalescent COVID-19 patients displaying time of blood collection (**d**), SARS-CoV-2-specific IgG antibody levels (**e**) and SARS-CoV-2-specific IgA antibody levels (**f**), as determined by ELISA. Data are displayed as mean ± SD. Asterisks indicate significant differences between groups. * $P < 0.05$, ** $P < 0.01$, *** $P < 0.001$, **** $P < 0.0001$, ns = not significant.

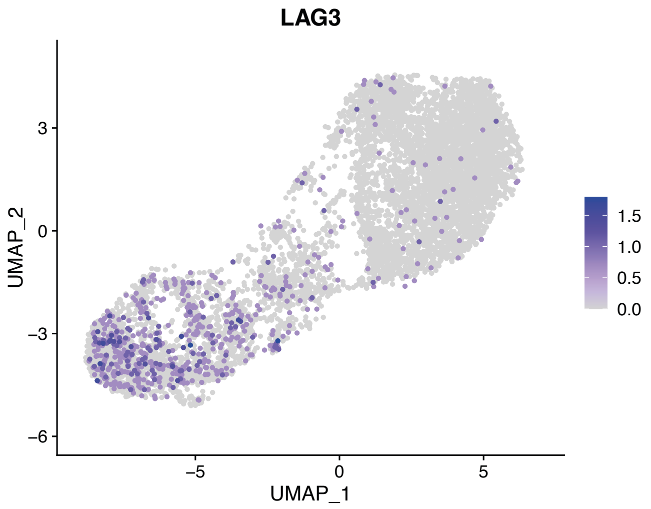

Supplemental Figure 2. Exhausted CD8+ T cells predominantly localize to the effector CD8+ T cell region. UMAP plot shows expression of the LAG-3 exhaustion marker by individual CD8+ T cells from all patients.

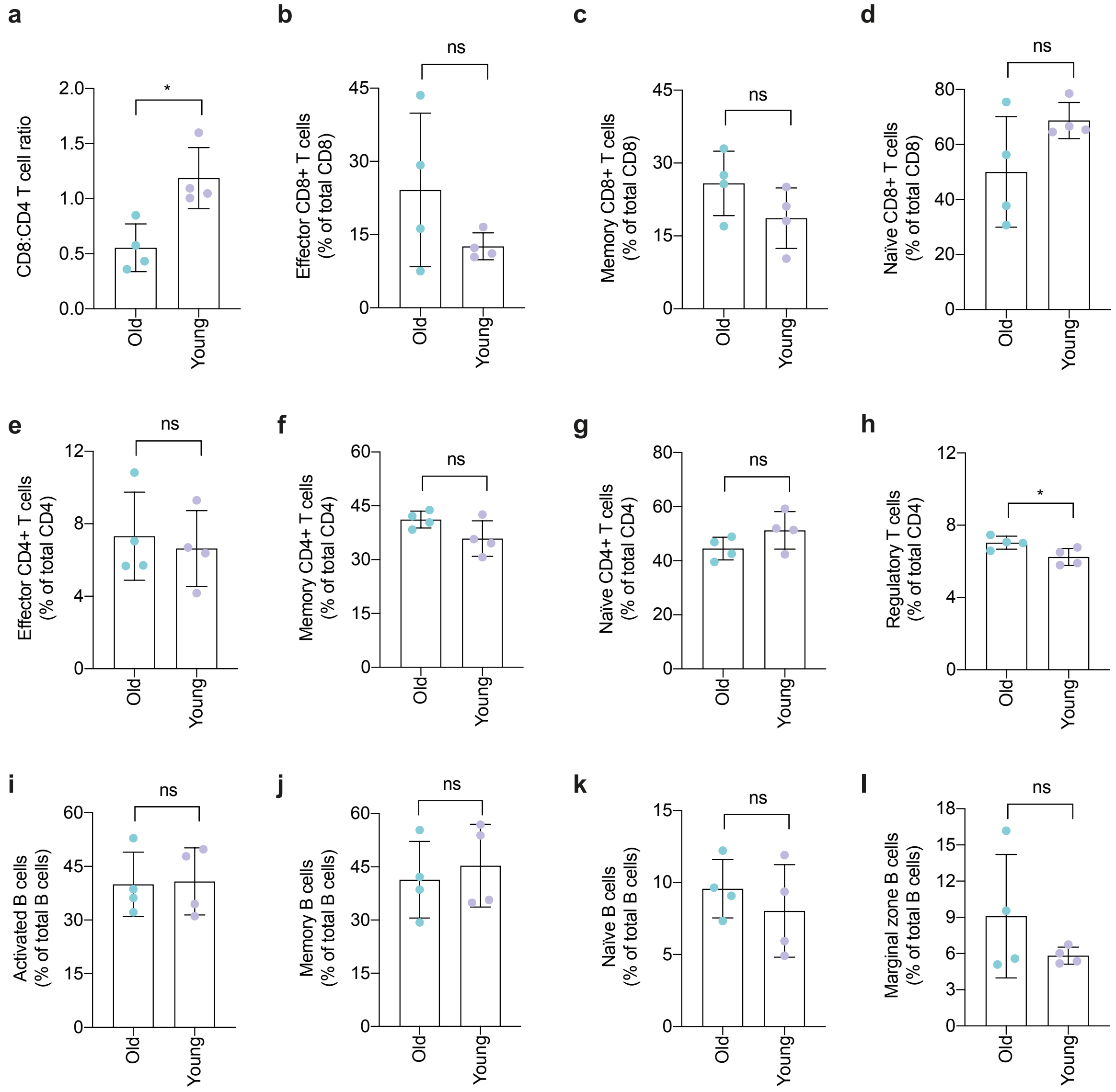

Supplemental Figure 3. Proportions of lymphocyte subsets across studied age groups. Bar graphs compare the proportions of T cell and B cell subsets in the old and young groups. Data are displayed as mean ± SD. Asterisks indicate significant differences between groups. * $P < 0.05$, ** $P < 0.01$, *** $P < 0.001$, **** $P < 0.0001$, ns = not significant.

Bieberich et al., Single-cell atlas of lymphocytes in COVID-19 patients

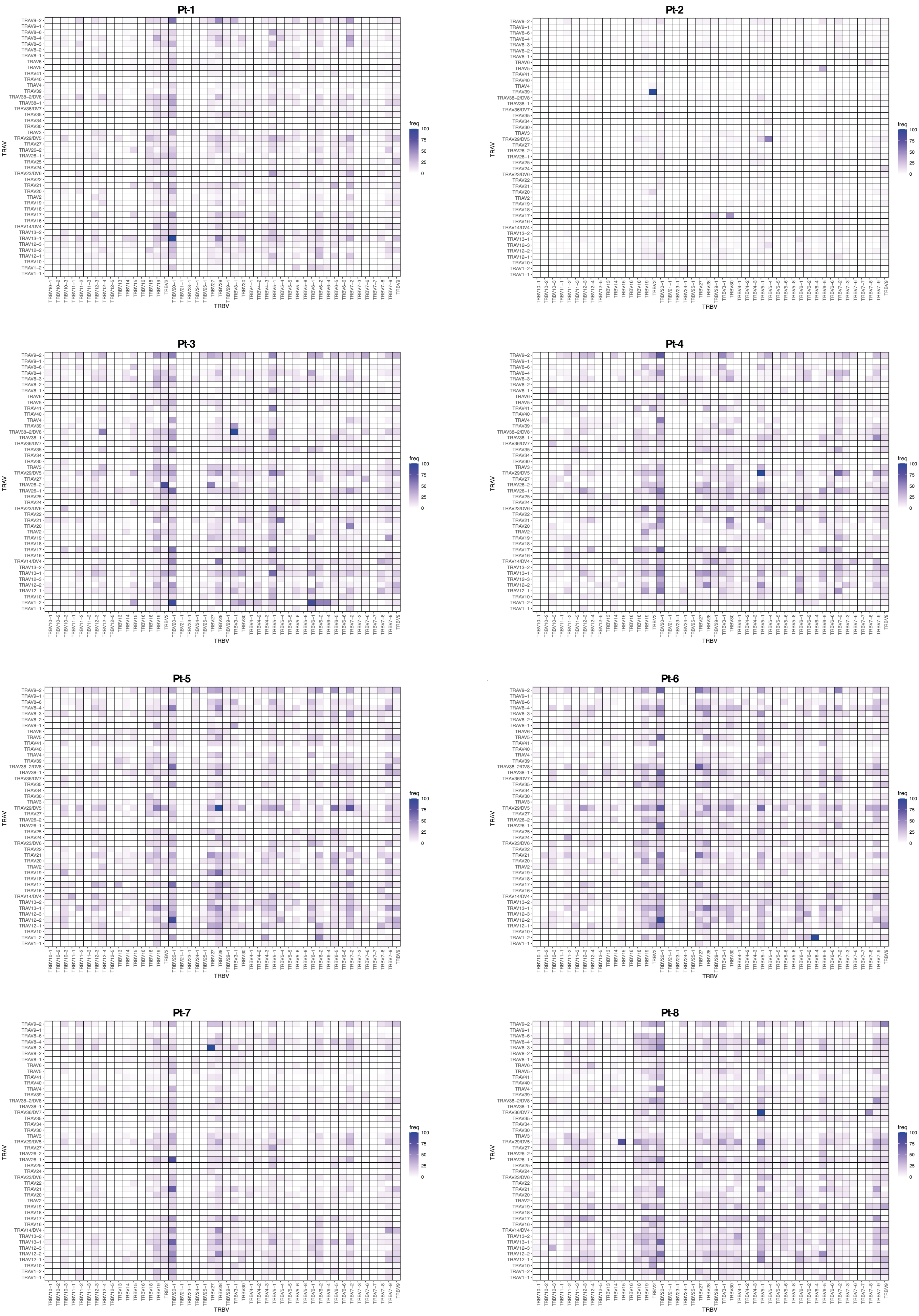

Supplemental Figure 4. **TCR V-gene pairings in convalescent COVID-19 patients.** Heatmaps display the relative frequencies of specific TCR V-gene pairings in the T cells of individual patients. Data is normalized to the most frequent pairing found in each patient.

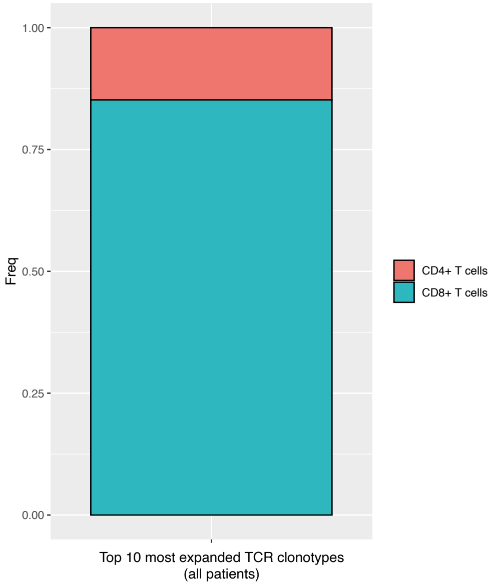

Supplemental Figure 5. **T cell clonal expansion in convalescent COVID-19 patients is dominated by CD8+ subsets**. Bar graph shows the proportions of CD8+ T cells versus CD4+ T cells within the top ten most expanded TCR clonotypes from each patient.

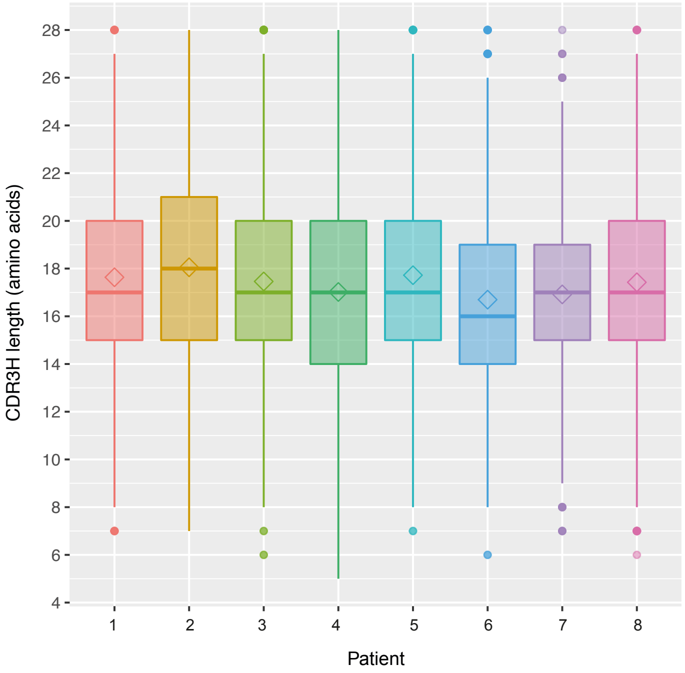

Supplemental Figure 6. BCR heavy chain CDR3 length distribution in convalescent COVID-19 patients.

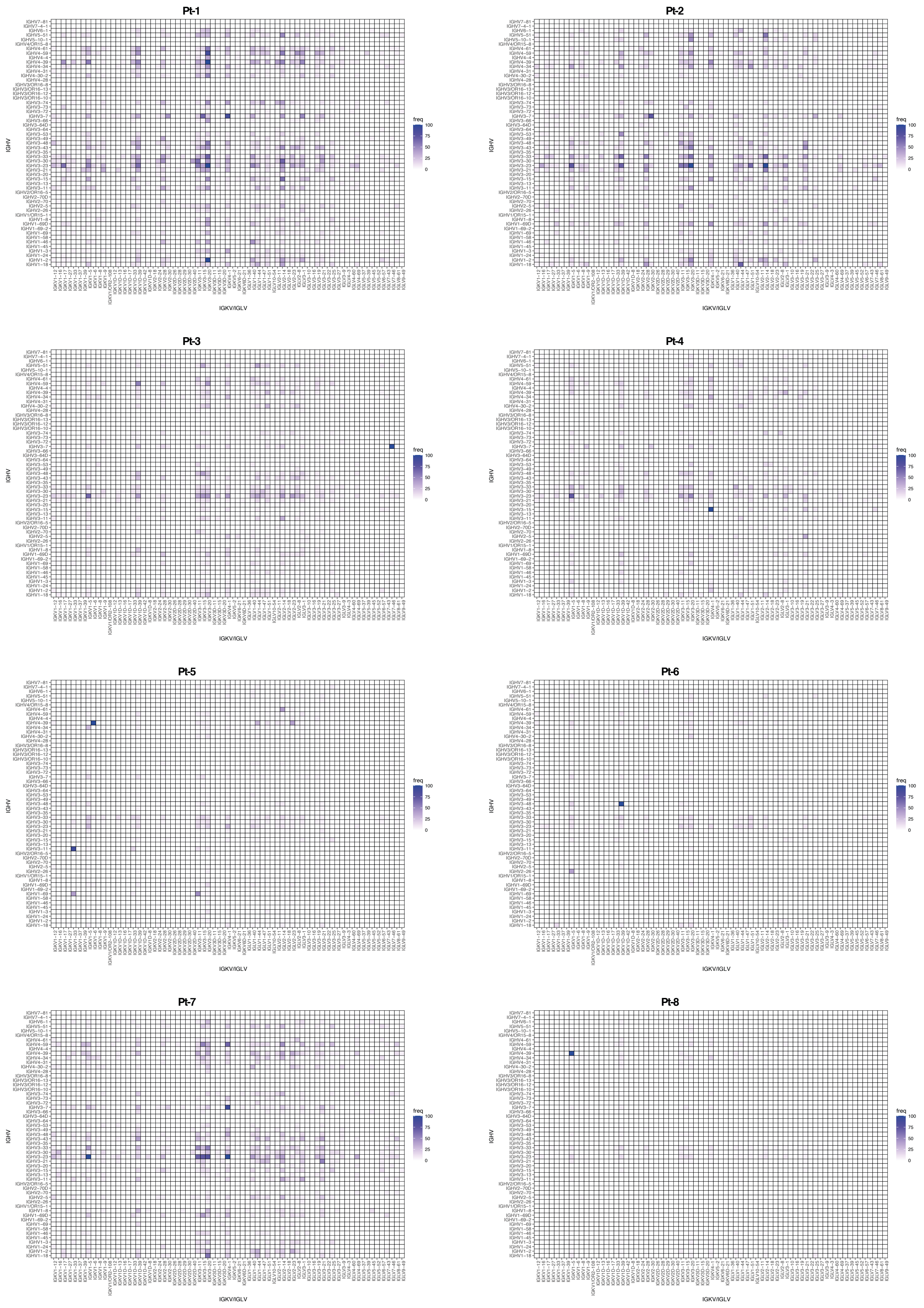
Pt-1
Pt-2
Pt-3
Pt-4
Pt-5
Pt-6
Pt-7
Pt-8
IGHV
IGKV/IGLV
freq
100
75
50
25
0

Supplemental Figure 7. **BCR V-gene pairings in convalescent COVID-19 patients.** Heatmaps display the relative frequencies of specific BCR V-gene pairings in the B cells of individual patients. Data is normalized to the most frequent pairing found in each patient.

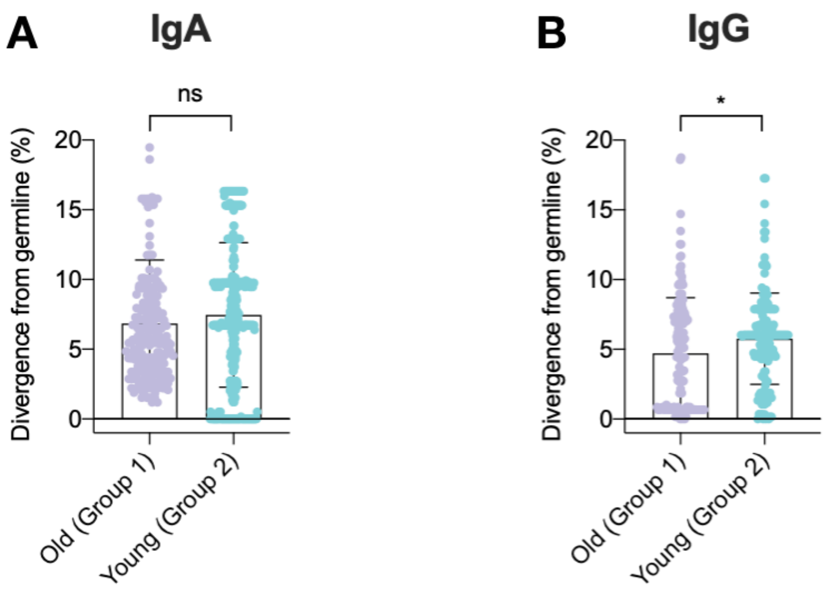

Supplemental Figure 8. SHM levels of isotype specific clonally expanded (>1 cell) cells. All SHM levels were calculated from cells that are part of clonotypes, which are composed of >1 cell. **a**, Boxplots display SHM levels in clonally expanded (>1) B cells that are IgG+, of old and young patients. **b**, Boxplots display SHM levels in clonally expanded (>1) B cells that are IgA+, of old and young patients. Each dot represents one cell.

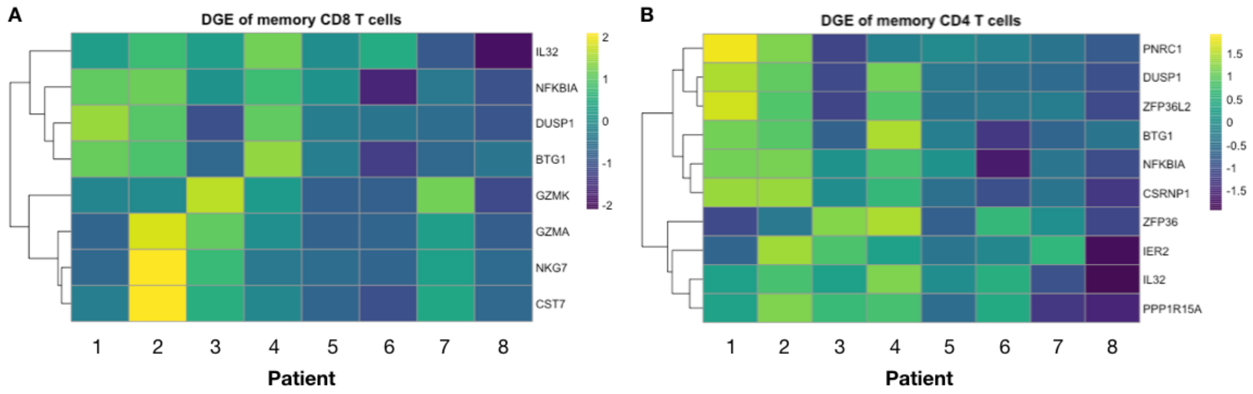

Supplemental Figure 9. Differential gene expression analysis of memory T cells across patients. a, Heatmap shows differential gene expression (DGE) of CD8+ memory T cells. **b**, Heatmap shows differential gene expression (DGE) of CD4+ memory T cells. Genes were filtered to include those with a minimum 50% fold-change in expression level between groups.

Supplemental Table 1. Characteristics of convalescent COVID-19 patients analyzed in this study

Patient ID	Age	Sex	BMI (kg/m^2)	RT-PCR test	Days ill (before PCR / after PCR / total)	Restrictions	Sample collection (days after symptom onset)	Rapid test (LFA)	ELISA test	IgA titer (OD ratio)	IgG titer (OD ratio)
Pt-1	59	F	33.9	Positive	7 / 7 / 14	Bedridden	32	IgM+ / IgG+	IgA+ / IgG+	10.8	13.1
Pt-2	63	M	24.4	Positive	7 / 7 / 14	Bedridden	41	IgM+ / IgG+	IgA+ / IgG+	13.5	12.8
Pt-3	74	F	23.4	Positive	2 / 7 / 9	Help needed	32	IgM+ / IgG+	IgA+ / IgG+	8.1	9.1
Pt-4)	71	F	28.9	Positive	5 / 7 / 12	Bedridden	30	IgM+ / IgG+	IgA+ / IgG+	9.9	12.4
Pt-5	37	M	28.7	Positive	2 / 5 / 7	No restrictions	33	IgM- / IgG+	IgA+ / IgG+	7.7	3.3
Pt-6	31	M	23.2	Positive	2 / 7 / 9	No restrictions	34	IgM- / IgG+	IgA+ / IgG+	6.5	8.5
Pt-7	23	F	22	Positive	0 / 7 / 7	No restrictions	39	IgM+ / IgG+	IgA+ / IgG+	4.8	5.7
Pt-8	33	M	26	Positive	2 / 2 / 4	No restrictions	30	IgM+ / IgG+	IgA+ / IgG+	4.1	9.5

Supplemental Table 2. HLA class I alleles of studied convalescent COVID-19 patients

Patient	HLA-A	HLA-B	HLA-Cw
Pt-1	A*03:01, A*30:01	B*13:02, B*40:02	C*02:02, C*06:02
Pt-2	A*02:01, A*26:01	B*38:01, B*44:02	C*05:01, C*12:03
Pt-3	A*01:01, A*24:02	B*07:02, B*15:29	C*07:02, C*07:04
Pt-4	A*02:01, A*02:01	B*15:01, B*35:01	C*03:04, C*04:01
Pt-5	A*11:01, A*11:01	B*18:03, B*40:01	C*03:04, C*07:01
Pt-6	A*01:01, A*68:01	B*07:02, B*35:03	C*04:01, C*07:02
Pt-7	A*03:01, A*24:02	B*18:01, B*35:02	C*04:01, C*07:01
Pt-8	A*02:01, A*24:02	B*13:02, B*35:02	C*04:01, C*06:02

Supplemental Table 3. GLIPH2 analysis of patient TCRs and known HLA-A*02:01-restricted SARS-CoV-2-specific TCRs

Cluster	Motif	CDR3b	TRBV	TRBJ	CDR3a	TRAV	TRAJ	Clone	Patient
1	QNTG	CASSmaraqNTGELFF	TRBV6-6	TRBJ2-2	CAGSIGFGNVLHC	TRAV25	TRAJ35	1059	Pt-02
1	QNTG	CASrdsqNTGELFF	TRBV25-1	TRBJ2-2	CAVNSYGGSQGNLIF	TRAV12-2	TRAJ42	20	Pt-08
1	QNTG	CASidqNTGELFF	TRBV5-1	TRBJ2-2	NA	NA	NA	NA	NA
1	QNTG	CASmdqNTGELFF	TRBV2	TRBJ2-2	NA	NA	NA	NA	NA
1	QNTG	CASSYqNTGELFF	TRBV7-8	TRBJ2-2	NA	NA	NA	NA	NA
1	QNTG	CASqdqNTGELFF	TRBV19	TRBJ2-2	NA	NA	NA	NA	NA
1	QNTG	CASSfqNTGELFF	TRBV7-8	TRBJ2-2	NA	NA	NA	NA	NA
1	QNTG	CAvLAeqNTGELFF	TRBV10-2	TRBJ2-2	NA	NA	NA	NA	NA
1	QNTG	CASnnqNTGELFF	TRBV7-8	TRBJ2-2	NA	NA	NA	NA	NA
1	QNTG	CASGtqNTGELFF	TRBV5-1	TRBJ2-2	NA	NA	NA	NA	NA
1	QNTG	CASlsqNTGELFF	TRBV7-8	TRBJ2-2	NA	NA	NA	NA	NA
1	QNTG	CATqvdqNTGELFF	TRBV5-1	TRBJ2-2	NA	NA	NA	NA	NA
1	QNTG	CSVEeqNTGELFF	TRBV29-1	TRBJ2-2	NA	NA	NA	NA	NA
1	QNTG	CARgdqNTGELFF	TRBV7-8	TRBJ2-2	NA	NA	NA	NA	NA
1	QNTG	CASSSqNTGELFF	TRBV7-8	TRBJ2-2	NA	NA	NA	NA	NA
1	QNTG	CASGrqNTGELFF	TRBV5-1	TRBJ2-2	NA	NA	NA	NA	NA
1	QNTG	CAptpdqNTGELFF	TRBV12-3	TRBJ2-2	NA	NA	NA	NA	NA
1	QNTG	CASnsqNTGELFF	TRBV12-3	TRBJ2-2	NA	NA	NA	NA	NA
1	QNTG	CASSVqNTGELFF	TRBV7-8	TRBJ2-2	NA	NA	NA	NA	NA

1	QNTG	CSARdeaGGQNTGELFF	TRBV20-1	TRBJ2-2	NA	NA	NA	NA	NA
1	QNTG	CSARdqpGGQNTGELFF	TRBV20-1	TRBJ2-2	NA	NA	NA	NA	NA
1	QNTG	CSARdqaaqNTGELFF	TRBV20-1	TRBJ2-2	NA	NA	NA	NA	NA
1	QNTG	CSARdLAaqNTGELFF	TRBV20-1	TRBJ2-2	NA	NA	NA	NA	NA
1	QNTG	CSARdrLAqNTGELFF	TRBV20-1	TRBJ2-2	NA	NA	NA	NA	NA
1	QNTG	CSARsrQGqNTGELFF	TRBV20-1	TRBJ2-2	NA	NA	NA	NA	NA
1	QNTG	CSARgesdqNTGELFF	TRBV20-1	TRBJ2-2	NA	NA	NA	NA	NA
1	QNTG	CASSDttrqNTGELFF	TRBV2	TRBJ2-2	NA	NA	NA	NA	NA
1	QNTG	CASrsrveqNTGELFF	TRBV2	TRBJ2-2	NA	NA	NA	NA	NA
1	QNTG	CSARgGLsqNTGELFF	TRBV20-1	TRBJ2-2	NA	NA	NA	NA	NA
1	QNTG	CSARgGQGqNTGELFF	TRBV20-1	TRBJ2-2	NA	NA	NA	NA	NA
1	QNTG	CSARdGLAqNTGELFF	TRBV20-1	TRBJ2-2	NA	NA	NA	NA	NA
1	QNTG	CASrGLAeqNTGELFF	TRBV2	TRBJ2-2	NA	NA	NA	NA	NA
1	QNTG	CASrdihqqNTGELFF	TRBV2	TRBJ2-2	NA	NA	NA	NA	NA
1	QNTG	CSARdsraqNTGELFF	TRBV20-1	TRBJ2-2	NA	NA	NA	NA	NA
1	QNTG	CSARdDRaqNTGELFF	TRBV20-1	TRBJ2-2	NA	NA	NA	NA	NA
1	QNTG	CSARdaeGGQNTGELFF	TRBV20-1	TRBJ2-2	NA	NA	NA	NA	NA
3	DIE	CASSLadierETQYF	TRBV11-2	TRBJ2-5	CAVRDQAGTALIF	TRAV2	TRAJ15	1420	Pt-08
3	DIE	CASirdieiNEQFF	TRBV28	TRBJ2-1	CVVFNQAGTALIF	TRAV12-1	TRAJ15	2740	Pt-08
3	DIE	CASSLdietYF	TRBV7-9	TRBJ2-7	NA	NA	NA	NA	NA
3	DIE	CASSwdiEAFF	TRBV7-9	TRBJ1-1	NA	NA	NA	NA	NA
3	DIE	CASTrdiEAFF	TRBV7-9	TRBJ1-1	NA	NA	NA	NA	NA

3	DIE	CASSVdiEAFF	TRBV7-9	TRBJ1-1	NA	NA	NA	NA	NA
3	DIE	CASTqdiEAFF	TRBV7-9	TRBJ1-1	NA	NA	NA	NA	NA
3	DIE	CASStdiEAFF	TRBV7-9	TRBJ1-1	NA	NA	NA	NA	NA
3	DIE	CASSSdiEQFF	TRBV7-9	TRBJ2-1	NA	NA	NA	NA	NA
3	DIE	CASSSdiEAFF	TRBV7-9	TRBJ1-1	NA	NA	NA	NA	NA
3	DIE	CASSDdiEAFF	TRBV7-9	TRBJ1-1	NA	NA	NA	NA	NA
3	DIE	CASSQdiEQYF	TRBV7-9	TRBJ2-7	NA	NA	NA	NA	NA
3	DIE	CASSQdiEAFF	TRBV7-9	TRBJ1-1	NA	NA	NA	NA	NA
3	DIE	CASSPdiEQFF	TRBV7-9	TRBJ2-1	NA	NA	NA	NA	NA
3	DIE	CASSPdiEQYF	TRBV7-9	TRBJ2-7	NA	NA	NA	NA	NA
3	DIE	CASSPdiEAFF	TRBV7-9	TRBJ1-1	NA	NA	NA	NA	NA
3	DIE	CASSadiEQFF	TRBV7-9	TRBJ2-1	NA	NA	NA	NA	NA
3	DIE	CASSPdiedFF	TRBV7-9	TRBJ1-1	NA	NA	NA	NA	NA
3	DIE	CASSLdiEAFF	TRBV7-9	TRBJ1-1	NA	NA	NA	NA	NA
6	DLNT	CASkrdlNTEAFF	TRBV5-5	TRBJ1-1	CAALLGPDKLIF	TRAV1-1	TRAJ34	1310	Pt-08
6	DLNT	CASDRGdlNTGELFF	TRBV19	TRBJ2-2	CAVRLTGGGNKLTF	TRAV12-2	TRAJ10	2520	Pt-08
6	DLNT	CASSSdlNTGELFF	TRBV11-2	TRBJ2-2	CALYSGAGSYQLTF	TRAV9-2	TRAJ28	2718	Pt-08
6	DLNT	CAItdlNTGELFF	TRBV27	TRBJ2-2	NA	NA	NA	NA	NA
6	DLNT	CAGQdlNTGELFF	TRBV7-2	TRBJ2-2	NA	NA	NA	NA	NA
6	DLNT	CASSDlNTGELFF	TRBV2	TRBJ2-2	NA	NA	NA	NA	NA
6	DLNT	CATedlNTGELFF	TRBV6-5	TRBJ2-2	NA	NA	NA	NA	NA
6	DLNT	CASndlNTGELFF	TRBV7-8	TRBJ2-2	NA	NA	NA	NA	NA

6	DLNT	CASndlNTGELFF	TRBV2	TRBJ2-2	NA	NA	NA	NA	NA
6	DLNT	CAlgdlNTGELFF	TRBV7-2	TRBJ2-2	NA	NA	NA	NA	NA
6	DLNT	CApgdlNTGELFF	TRBV7-2	TRBJ2-2	NA	NA	NA	NA	NA
6	DLNT	CASqdlNTGELFF	TRBV19	TRBJ2-2	NA	NA	NA	NA	NA
6	DLNT	CASqdlNTGELFF	TRBV10-1	TRBJ2-2	NA	NA	NA	NA	NA
6	DLNT	CAIqdlNTGELFF	TRBV13	TRBJ2-2	NA	NA	NA	NA	NA
6	DLNT	CATtdlNTGELFF	TRBV15	TRBJ2-2	NA	NA	NA	NA	NA
6	DLNT	CASydlNTGELFF	TRBV5-1	TRBJ2-2	NA	NA	NA	NA	NA
6	DLNT	CATlvdlNTGELFF	TRBV12-3	TRBJ2-2	NA	NA	NA	NA	NA
6	DLNT	CSAhgdlNTGELFF	TRBV20-1	TRBJ2-2	NA	NA	NA	NA	NA
6	DLNT	CASTdlNTGELFF	TRBV5-4	TRBJ2-2	NA	NA	NA	NA	NA
6	DLNT	CASTdlNTGELFF	TRBV6-5	TRBJ2-2	NA	NA	NA	NA	NA
6	DLNT	CAardqrdlNTGELFF	TRBV2	TRBJ2-2	NA	NA	NA	NA	NA
38	SPD%E	CASSPdNEQFF	TRBV7-6	TRBJ2-1	CAVQAATGRRALTF	TRAV20	TRAJ5	375	Pt-04
38	SPD%E	CASSPdsEQYF	TRBV7-9	TRBJ2-7	NA	NA	NA	NA	NA
38	SPD%E	CASSPdiEQFF	TRBV7-9	TRBJ2-1	NA	NA	NA	NA	NA
38	SPD%E	CASSPdiEQYF	TRBV7-9	TRBJ2-7	NA	NA	NA	NA	NA
38	SPD%E	CASSPdiEAFF	TRBV7-9	TRBJ1-1	NA	NA	NA	NA	NA
38	SPD%E	CASSPdiedFF	TRBV7-9	TRBJ1-1	NA	NA	NA	NA	NA
38	SPD%E	CASSPdgEQYF	TRBV7-9	TRBJ2-7	NA	NA	NA	NA	NA
44	SDLD	CASSDlDRaGANVLTF	TRBV6-4	TRBJ2-6	CAVRDKPRLMF	TRAV3	TRAJ31	1849	Pt-02
44	SDLD	CAISdlDRGilETQYF	TRBV10-3	TRBJ2-5	CAVKTYGQNFVF	TRAV8-1	TRAJ26	723	Pt-04

44	SDLD	CASSDldGGEAFF	TRBV2	TRBJ1-1	NA	NA	NA	NA	NA
44	SDLD	CATSDLdsGELFF	TRBV24-1	TRBJ2-2	NA	NA	NA	NA	NA
44	SDLD	CASSDldnlvAFF	TRBV2	TRBJ1-1	NA	NA	NA	NA	NA
44	SDLD	CASSDldTGELFF	TRBV2	TRBJ2-2	NA	NA	NA	NA	NA
48	EQNT	CASSQeqNTEAFF	TRBV3-1	TRBJ1-1	CAVGAPGAGSYQLTF	TRAV8-3	TRAJ28	3180	Pt-08
48	EQNT	CAvLAeqNTGELFF	TRBV10-2	TRBJ2-2	NA	NA	NA	NA	NA
48	EQNT	CSVEeqNTGELFF	TRBV29-1	TRBJ2-2	NA	NA	NA	NA	NA
48	EQNT	CASrsrveqNTGELFF	TRBV2	TRBJ2-2	NA	NA	NA	NA	NA
48	EQNT	CASrGLAeqNTGELFF	TRBV2	TRBJ2-2	NA	NA	NA	NA	NA
59	DEVA	CATSDevavGELFF	TRBV24-1	TRBJ2-2	CAARRSQGNLIF	TRAV8-6	TRAJ42	2090	Pt-08
59	DEVA	CASSQdevasGELFF	TRBV4-1	TRBJ2-2	CAVSPAALGFGNVLHC	TRAV3	TRAJ35	2652	Pt-08
59	DEVA	CSARdevahNTGELFF	TRBV20-1	TRBJ2-2	NA	NA	NA	NA	NA
61	SLD%E	CASSLdwEQYF	TRBV13	TRBJ2-7	CIVRHSGGYQKVTF	TRAV26-1	TRAJ13	922	Pt-02
61	SLD%E	CASSLdTEAFF	TRBV12-4	TRBJ1-1	CAPGWAGTALIF	TRAV6	TRAJ15	8	Pt-08
61	SLD%E	CASSLdietYF	TRBV7-9	TRBJ2-7	NA	NA	NA	NA	NA
61	SLD%E	CASSLdsEQFF	TRBV7-9	TRBJ2-1	NA	NA	NA	NA	NA
61	SLD%E	CASSLdvEQYF	TRBV7-9	TRBJ2-7	NA	NA	NA	NA	NA
61	SLD%E	CASSLdiEAFF	TRBV7-9	TRBJ1-1	NA	NA	NA	NA	NA
79	S%ANTGE	CASSYaNTGELFF	TRBV5-4	TRBJ2-2	CAVGVDSNYQLIW	TRAV39	TRAJ33	1104	Pt-04
79	S%ANTGE	CASSEaNTGELFF	TRBV7-9	TRBJ2-2	NA	NA	NA	NA	NA
79	S%ANTGE	CASSDaNTGELFF	TRBV5-1	TRBJ2-2	NA	NA	NA	NA	NA
79	S%ANTGE	CASSnaNTGELFF	TRBV7-9	TRBJ2-2	NA	NA	NA	NA	NA

82	S%GTGHQP	CATSkGTGhQPQHF	TRBV15	TRBJ1-5	CVVRGSNDYKLSF	TRAV12-1	TRAJ20	827	Pt-08
82	S%GTGHQP	CATSgGTGhQPQHF	TRBV24-1	TRBJ1-5	NA	NA	NA	NA	NA
82	S%GTGHQP	CASSLGTGhQPQHF	TRBV27	TRBJ1-5	NA	NA	NA	NA	NA
98	SIIG	CASSIigNQPQHF	TRBV19	TRBJ1-5	CILNSGGSNYKLTF	TRAV26-2	TRAJ53	949	Pt-02
98	SIIG	CASSQsiigGTETQYF	TRBV16	TRBJ2-5	CAEKGDKIIF	TRAV5	TRAJ30	547	Pt-04
98	SIIG	CASSIiGGgNTGELFF	TRBV19	TRBJ2-2	NA	NA	NA	NA	NA
102	SKGN	CASSYskgNQPQHF	TRBV6-6	TRBJ1-5	CILSRNFGNEKLTF	TRAV26-2	TRAJ48	1621	Pt-04
102	SKGN	CAISEskGNTIYF	TRBV10-3	TRBJ1-3	CVVSADNYGQNFVF	TRAV10	TRAJ26	1445	Pt-08
102	SKGN	CSARdskgngwNTGELFF	TRBV20-1	TRBJ2-2	NA	NA	NA	NA	NA
166	SLGTG%D	CASSLGTGdddTF	TRBV7-9	TRBJ1-2	CALLGDSNYQLIW	TRAV16	TRAJ33	418	Pt-08
166	SLGTG%D	CASSLGTGGdLHF	TRBV7-9	TRBJ1-6	NA	NA	NA	NA	NA
181	SDGTS%E	CAISdGTSYEQYF	TRBV10-3	TRBJ2-7	CAGPGVSSGGSNYKLTF	TRAV25	TRAJ53	3305	Pt-08
181	SDGTS%E	CASSDGTSnEQYF	TRBV5-1	TRBJ2-7	NA	NA	NA	NA	NA
192	SGGTG%QP	CASSgGTGGQPQHF	TRBV2	TRBJ1-5	CVVNSDYGQNFVF	TRAV12-1	TRAJ26	1429	Pt-08
192	SGGTG%QP	CATSgGTGhQPQHF	TRBV24-1	TRBJ1-5	NA	NA	NA	NA	NA
202	SFLAGG%TGE	CASSfLAGGqTGELFF	TRBV5-6	TRBJ2-2	CAVNKNTGGFKTIF	TRAV8-1	TRAJ9	1869	Pt-08
202	SFLAGG%TGE	CASSfLAGGNTGELFF	TRBV19	TRBJ2-2	NA	NA	NA	NA	NA
247	SD%YG	CASSDdYGYTF	TRBV5-1	TRBJ1-2	CAVNAQGNNRLAF	TRAV8-1	TRAJ7	1898	Pt-02
247	SD%YG	CASSDsYGYTF	TRBV7-8	TRBJ1-2	NA	NA	NA	NA	NA
259	SY%NTGE	CASSYaNTGELFF	TRBV5-4	TRBJ2-2	CAVGVDSNYQLIW	TRAV39	TRAJ33	1104	Pt-04
259	SY%NTGE	CASSYqNTGELFF	TRBV7-8	TRBJ2-2	NA	NA	NA	NA	NA
279	%YANTGE	CASSYaNTGELFF	TRBV5-4	TRBJ2-2	CAVGVDSNYQLIW	TRAV39	TRAJ33	1104	Pt-04

279	%YANTGE	CAIqyaNTGELFF	TRBV15	TRBJ2-2	NA	NA	NA	NA	NA
284	QQAN	CASSQqaNTEAFF	TRBV3-1	TRBJ1-1	CAVTYTGANSKLTF	TRAV8-6	TRAJ56	3280	Pt-08
284	QQAN	CASqqaNTGELFF	TRBV19	TRBJ2-2	NA	NA	NA	NA	NA
285	S%TNTGE	CASSGTNTGELFF	TRBV6-5	TRBJ2-2	CAASSSGTYKYIF	TRAV29/DV5	TRAJ40	782	Pt-02
285	S%TNTGE	CASSDtNTGELFF	TRBV5-1	TRBJ2-2	NA	NA	NA	NA	NA
285	S%TNTGE	CASShtNTGELFF	TRBV9	TRBJ2-2	NA	NA	NA	NA	NA
288	DLNS	CAGGgdlnSGNTIYF	TRBV12-3	TRBJ1-3	CVGNSGYALNF	TRAV12-1	TRAJ41	480	Pt-08
288	DLNS	CASSDlnSGEQYF	TRBV2	TRBJ2-7	NA	NA	NA	NA	NA
288	DLNS	CATmddlnsGELFF	TRBV2	TRBJ2-2	NA	NA	NA	NA	NA
302	%TENTGE	CAltteNTGELFF	TRBV30	TRBJ2-2	CVVNKITGTASKLTF	TRAV12-1	TRAJ44	3701	Pt-08
302	%TENTGE	CATSteNTGELFF	TRBV15	TRBJ2-2	NA	NA	NA	NA	NA
385	SPLAGG%TGE	CASSPLAGGtTGELFF	TRBV13	TRBJ2-2	CAMREGGNSGGSNYKLTF	TRAV14/DV4	TRAJ53	1808	Pt-08
385	SPLAGG%TGE	CASSPLAGGNTGELFF	TRBV3-1	TRBJ2-2	NA	NA	NA	NA	NA
385	SPLAGG%TGE	CASSPLAGGNTGELFF	TRBV4-3	TRBJ2-2	NA	NA	NA	NA	NA
428	%GTNTGE	CASSGTNTGELFF	TRBV6-5	TRBJ2-2	CAASSSGTYKYIF	TRAV29/DV5	TRAJ40	782	Pt-02
428	%GTNTGE	CASGGTNTGELFF	TRBV2	TRBJ2-2	NA	NA	NA	NA	NA
533	SPDS%	CASSPdSPLHF	TRBV6-6	TRBJ1-6	CALSEARNFNKFYF	TRAV19	TRAJ21	3176	Pt-08
533	SPDS%	CASSPdsEQYF	TRBV7-9	TRBJ2-7	NA	NA	NA	NA	NA
540	S%GTSNE	CASSfGTSNEQFF	TRBV27	TRBJ2-1	CAETLAGGSTLGRLYF	TRAV5	TRAJ18	448	Pt-08
540	S%GTSNE	CASSDGTSnEQYF	TRBV5-1	TRBJ2-7	NA	NA	NA	NA	NA
580	SL%VE	CSgslgvEQYF	TRBV20-1	TRBJ2-7	CAVNFYNQGGKLIF	TRAV8-1	TRAJ23	610	Pt-08
580	SL%VE	CASSLdvEQYF	TRBV7-9	TRBJ2-7	NA	NA	NA	NA	NA

618	RD%DT	CASrdaDTQYF	TRBV5-5	TRBJ2-3	CVVNTGFQKLVF	TRAV12-1	TRAJ8	2922	Pt-08
618	RD%DT	CANrdTDTQYF	TRBV3-1	TRBJ2-3	NA	NA	NA	NA	NA
664	SG%NTGE	CASSGTNTGELFF	TRBV6-5	TRBJ2-2	CAASSSGTYKYIF	TRAV29/DV5	TRAJ40	782	Pt-02
664	SG%NTGE	CASSGLNTGELFF	TRBV5-1	TRBJ2-2	NA	NA	NA	NA	NA

Supplemental Table 4. GLIPH2 analysis of patient TCRs and known HLA-A*02:01-restricted CMV- and EBV-specific TCRs

Specificity group	Motif	CDR3b	TRBV	TRBJ	CDR3a	TRAV	TRAJ	Patient
15	SSA%YG	CASSSASYGYTF	TRBV19	TRBJ1-2	NA	NA	NA	NLVPMVATV_pp65_CMV
15	SSA%YG	CASSSANYGYTF	TRBV12-4	TRBJ1-2	CAGPMKTSYDKVIF	TRAV35	TRAJ50	Pt-08
15	SSA%YG	CASSSANYGYTF	TRBV12-4	TRBJ1-2	NA	NA	NA	NLVPMVATV_pp65_CMV
15	SSA%YG	CASSSANYGYTF	TRBV19	TRBJ1-2	NA	NA	NA	NLVPMVATV_pp65_CMV
15	SSA%YG	CASSSANYGYTF	TRBV12-3	TRBJ1-2	NA	NA	NA	NLVPMVATV_pp65_CMV
15	SSA%YG	CASSSANYGYTF	TRBV11-1	TRBJ1-2	NA	NA	NA	NLVPMVATV_pp65_CMV
15	SSA%YG	CASSSAYYGYTF	TRBV12-4	TRBJ1-2	NA	NA	NA	NLVPMVATV_pp65_CMV
15	SSA%YG	CASSSATYGYTF	TRBV19	TRBJ1-2	NA	NA	NA	NLVPMVATV_pp65_CMV
15	SSA%YG	CASSSAHYGYTF	TRBV11-1	TRBJ1-2	NA	NA	NA	NLVPMVATV_pp65_CMV
15	SSA%YG	CASSSAFYGYTF	TRBV19	TRBJ1-2	NA	NA	NA	NLVPMVATV_pp65_CMV
15	SSA%YG	CASSSAFYGYTF	TRBV11-1	TRBJ1-2	NA	NA	NA	NLVPMVATV_pp65_CMV
64	GQAW	CASSSPVQGQAWVYGYTF	TRBV28	TRBJ1-2	CAVRPADRGSTLGRLYF	TRAV1-2	TRAJ18	Pt-08
64	GQAW	CAISDPPGQAWGSPLHF	TRBV10-3	TRBJ1-6	CAARYGGATNKLIF	TRAV29/DV5	TRAJ32	Pt-08
64	GQAW	CASSFGQAWETQYF	TRBV7-9	TRBJ2-5	NA	NA	NA	NLVPMVATV_pp65_CMV
64	GQAW	CASSFGQAWYEQYF	TRBV7-9	TRBJ2-7	NA	NA	NA	NLVPMVATV_pp65_CMV
87	R%GVGNT	CSARTGVGNTIYF	TRBV20-1	TRBJ1-3	NA	NA	NA	GLCTLVAML_BMLF1_EBV
87	R%GVGNT	CSARSGVGNTIYF	TRBV20-1	TRBJ1-3	NA	NA	NA	GLCTLVAML_BMLF1_EBV
87	R%GVGNT	CSARVGVGNTIYF	TRBV20-1	TRBJ1-3	NA	NA	NA	GLCTLVAML_BMLF1_EBV

87	R%GVGNT	CASREGVGNTIYF	TRBV6-5	TRBJ1-3	CAMGGSEKLVF	TRAV29/DV5	TRAJ57	Pt-08
109	SL%LENE	CASSLGLENEQFF	TRBV7-8	TRBJ2-1	NA	NA	NA	GLCTLVAML_BMLF1_EBV
109	SL%LENE	CASSLTLENEQFF	TRBV7-3	TRBJ2-1	CAAKTGANNLFF	TRAV29/DV5	TRAJ36	Pt-04
187	QRGF	CASRSAQRGFTETQYF	TRBV28	TRBJ2-5	NA	NA	NA	NLVPMVATV_pp65_CMV
187	QRGF	CASSPSTQRGFTAEAFF	TRBV7-2	TRBJ1-1	CARNKGGTGFQKLVF	TRAV6	TRAJ8	Pt-02
198	SFGQA%YE	CASSFGQAWYEQYF	TRBV7-9	TRBJ2-7	NA	NA	NA	NLVPMVATV_pp65_CMV
198	SFGQA%YE	CASSFGQAHYEQYF	TRBV5-1	TRBJ2-7	CARLPWRDNYGQNFVF	TRAV12-3	TRAJ26	Pt-08
204	SLA%GATNEK	CASSLAPGATNEKLFF	TRBV7-6	TRBJ1-4	NA	NA	NA	NLVPMVATV_pp65_CMV
204	SLA%GATNEK	CASSLAPGATNEKLFF	TRBV11-1	TRBJ1-4	NA	NA	NA	NLVPMVATV_pp65_CMV
204	SLA%GATNEK	CASSLAPGATNEKLFF	TRBV7-9	TRBJ1-4	NA	NA	NA	NLVPMVATV_pp65_CMV
204	SLA%GATNEK	CASSLAGGATNEKLFF	TRBV5-1	TRBJ1-4	CAAKSGAGSYQLTF	TRAV29/DV5	TRAJ28	Pt-04
225	SLTTG%	CASSLTTGGYTF	TRBV19	TRBJ1-2	CAVGYDMRF	TRAV22	TRAJ43	Pt-04
225	SLTTG%	CASSLTTGIQYF	TRBV6-2	TRBJ2-4	NA	NA	NA	YVLDHLIVV_BRLF1_EBV
239	S%LLAGYNE	CASSLLLAGYNEQFF	TRBV11-2	TRBJ2-1	NA	NA	NA	NLVPMVATV_pp65_CMV
239	S%LLAGYNE	CASSQLLAGYNEQFF	TRBV4-3	TRBJ2-1	CAASSLSGGSNYKLTF	TRAV23/DV6	TRAJ53	Pt-04
292	SQ%PGGE	CASSQDPGGEQYF	TRBV4-3	TRBJ2-7	CAENGGTSYGKLTF	TRAV13-2	TRAJ52	Pt-02
292	SQ%PGGE	CASSQSPGGEQYF	TRBV14	TRBJ2-7	NA	NA	NA	GLCTLVAML_BMLF1_EBV
346	SLL%SNQP	CASSLLVSNQPQHF	TRBV2	TRBJ1-5	CAIVRDDKIIF	TRAV17	TRAJ30	Pt-04
346	SLL%SNQP	CASSLLGSNQPQHF	TRBV27	TRBJ1-5	CAVLPQGGSEKLVF	TRAV36/DV7	TRAJ57	Pt-04
346	SLL%SNQP	CASSLLGSNQPQHF	TRBV7-8	TRBJ1-5	NA	NA	NA	GLCTLVAML_BMLF1_EBV
372	SLY%DT	CASSLYPDTQYF	TRBV27	TRBJ2-3	CAARYSGGGADGLTF	TRAV13-1	TRAJ45	Pt-04
372	SLY%DT	CASSLYSDTQYF	TRBV28	TRBJ2-3	NA	NA	NA	YVLDHLIVV_BRLF1_EBV

433	SPGT%KET	CASSPGTDKETQYF	TRBV5-1	TRBJ2-5	CAVNRPGGGNKLTF	TRAV12-2	TRAJ10	Pt-08
433	SPGT%KET	CASSPGTLKETQYF	TRBV12-3	TRBJ2-5	NA	NA	NA	NLVPMVATV_pp65_CMV
441	S%VGGYE	CASSLVGGYEQYF	TRBV5-4	TRBJ2-7	CAIRRGGGTSYGKLTF	TRAV13-1	TRAJ52	Pt-08
441	S%VGGYE	CASSHVGGYEQYF	TRBV4-1	TRBJ2-7	NA	NA	NA	GLCTLVAML_BMLF1_EBV
472	SL%GSNQP	CASSLQGSNQPQHF	TRBV6-2	TRBJ1-5	NA	NA	NA	YVLDHLIVV_BRLF1_EBV
472	SL%GSNQP	CASSLEGSNQPQHF	TRBV7-8	TRBJ1-5	CDNAGNMLTF	TRAV27	TRAJ39	Pt-08
472	SL%GSNQP	CASSLLGSNQPQHF	TRBV27	TRBJ1-5	CAVLPQGGSEKLVF	TRAV36/DV7	TRAJ57	Pt-04
472	SL%GSNQP	CASSLLGSNQPQHF	TRBV7-8	TRBJ1-5	NA	NA	NA	GLCTLVAML_BMLF1_EBV
501	SLG%ENE	CASSLGLENEQFF	TRBV7-8	TRBJ2-1	NA	NA	NA	GLCTLVAML_BMLF1_EBV
501	SLG%ENE	CASSLGEENEQFF	TRBV7-9	TRBJ2-1	CAESIFYNAGNMLTF	TRAV5	TRAJ39	Pt-08
568	S%QGSNQP	CASSPQGSNQPQHF	TRBV18	TRBJ1-5	CAASAGNAGKSTF	TRAV29/DV5	TRAJ27	Pt-08
568	S%QGSNQP	CASSLQGSNQPQHF	TRBV6-2	TRBJ1-5	NA	NA	NA	YVLDHLIVV_BRLF1_EBV
592	S%TGTYE	CATSWTGTYEQYF	TRBV15	TRBJ2-7	NA	NA	NA	YVLDHLIVV_BRLF1_EBV
592	S%TGTYE	CASSLTGTYEQYF	TRBV11-2	TRBJ2-7	CAERMAGNMLTF	TRAV13-2	TRAJ39	Pt-04
592	S%TGTYE	CASSLTGTYEQYF	TRBV4-1	TRBJ2-7	CVILTGNQFYF	TRAV29/DV5	TRAJ49	Pt-08
595	S%GQQET	CASSYGQQETQYF	TRBV6-5	TRBJ2-5	CAVRAPGGKLIF	TRAV1-1	TRAJ23	Pt-08
595	S%GQQET	CASSFGQQETQYF	TRBV12-4	TRBJ2-5	NA	NA	NA	GLCTLVAML_BMLF1_EBV
600	SG%GGTDT	CASSGVGGTDTQYF	TRBV9	TRBJ2-3	NA	NA	NA	NLVPMVATV_pp65_CMV
600	SG%GGTDT	CATSGQGGTDTQYF	TRBV24-1	TRBJ2-3	CAGDDAGGTSYGKLTF	TRAV27	TRAJ52	Pt-02
647	SRGA%E	CAWSRGAVEQFF	TRBV30	TRBJ2-1	NA	NA	NA	GLCTLVAML_BMLF1_EBV
647	SRGA%E	CASSRGAYEQYF	TRBV14	TRBJ2-7	CATYGGATNKLIF	TRAV14/DV4	TRAJ32	Pt-04
648	SL%GYTE	CASSLGGYTEAFF	TRBV7-2	TRBJ1-1	CALSGRSGGYQKVTF	TRAV16	TRAJ13	Pt-02

648	SL%GYTE	CASSLEGYTEAFF	TRBV11-2	TRBJ1-1	NA	NA	NA	NLVPMVATV_pp65_CMV
648	SL%GYTE	CASSLEGYTEAFF	TRBV27	TRBJ1-1	NA	NA	NA	NLVPMVATV_pp65_CMV
680	S%ANYG	CASSFANYGYTF	TRBV19	TRBJ1-2	NA	NA	NA	NLVPMVATV_pp65_CMV
680	S%ANYG	CASSSANYGYTF	TRBV12-4	TRBJ1-2	CAGPMKTSYDKVIF	TRAV35	TRAJ50	Pt-08
680	S%ANYG	CASSSANYGYTF	TRBV12-4	TRBJ1-2	NA	NA	NA	NLVPMVATV_pp65_CMV
680	S%ANYG	CASSSANYGYTF	TRBV19	TRBJ1-2	NA	NA	NA	NLVPMVATV_pp65_CMV
680	S%ANYG	CASSSANYGYTF	TRBV12-3	TRBJ1-2	NA	NA	NA	NLVPMVATV_pp65_CMV
680	S%ANYG	CASSSANYGYTF	TRBV11-1	TRBJ1-2	NA	NA	NA	NLVPMVATV_pp65_CMV
689	S%GNTE	CASSLGNTEAFF	TRBV12-3	TRBJ1-1	CAVREGYSTLTF	TRAV1-1	TRAJ11	Pt-08
689	S%GNTE	CASSLGNTEAFF	TRBV6-2	TRBJ1-1	NA	NA	NA	GLCTLVAML_BMLF1_EBV
689	S%GNTE	CASSRGNTEAFF	TRBV12-4	TRBJ1-1	CILRDSGYSTLTF	TRAV26-2	TRAJ11	Pt-08
689	S%GNTE	CASSDGNTEAFF	TRBV11-3	TRBJ1-1	CAVRTGNQFYF	TRAV8-3	TRAJ49	Pt-02
708	S%SDT	CASSLSDTQYF	TRBV5-1	TRBJ2-3	CALSTQAAGNKLTF	TRAV9-2	TRAJ17	Pt-08
708	S%SDT	CASSTSDTQYF	TRBV25-1	TRBJ2-3	NA	NA	NA	GLCTLVAML_BMLF1_EBV

Supplemental Table 5. Primer sequences for HLA class I typing

Primer Name	Restriction site (HindIII or BamHI)	Index	Target specific sequence	Final sequence (5' - 3')
PCR1_fwd-1	TACGCCAAGCTT	ACGAGTGCGT	TGGCCCTGACCSAGACCTG	TACGCCAAGCTTACGAGTGCGTTGGCCCTGACCSAGACCTG
PCR1_fwd-2	TACGCCAAGCTT	ACGCTCGACA	TGGCCCTGACCSAGACCTG	TACGCCAAGCTTACGCTCGACATGGCCCTGACCSAGACCTG
PCR1_fwd-3	TACGCCAAGCTT	AGACGCACTC	TGGCCCTGACCSAGACCTG	TACGCCAAGCTTAGACGCACTCTGGCCCTGACCSAGACCTG
PCR1_fwd-4	TACGCCAAGCTT	AGCACTGTAG	TGGCCCTGACCSAGACCTG	TACGCCAAGCTTAGCACTGTAGTGGCCCTGACCSAGACCTG
PCR1_fwd-5	TACGCCAAGCTT	ATCAGACACG	TGGCCCTGACCSAGACCTG	TACGCCAAGCTTATCAGACACGTGGCCCTGACCSAGACCTG
PCR1_fwd-6	TACGCCAAGCTT	ATATCGCGAG	TGGCCCTGACCSAGACCTG	TACGCCAAGCTTATATCGCGAGTGGCCCTGACCSAGACCTG
PCR1_fwd-7	TACGCCAAGCTT	CGTGTCTCTA	TGGCCCTGACCSAGACCTG	TACGCCAAGCTTCGTGTCTCTATGGCCCTGACCSAGACCTG
PCR1_fwd-8	TACGCCAAGCTT	CTCGCGTGTC	TGGCCCTGACCSAGACCTG	TACGCCAAGCTTCTCGCGTGTCTGGCCCTGACCSAGACCTG
PCR1_rev	ACCCGGGGATCC	-	GKCCTCGCTCTGGTTGTAGT	ACCCGGGGATCCGKCCTCGCTCTGGTTGTAGT
PCR2_fwd-1	TACGCCAAGCTT	TAGTATCAGC	ACTACAACCAGAGCGAGGMC	TACGCCAAGCTTTAGTATCAGCACTACAACCAGAGCGAGGMC
PCR2_fwd-2	TACGCCAAGCTT	TCTCTATGCG	ACTACAACCAGAGCGAGGMC	TACGCCAAGCTTTCTCTATGCGACTACAACCAGAGCGAGGMC
PCR2_fwd-3	TACGCCAAGCTT	TGATACGTCT	ACTACAACCAGAGCGAGGMC	TACGCCAAGCTTTGATACGTCTACTACAACCAGAGCGAGGMC
PCR2_fwd-4	TACGCCAAGCTT	TACTGAGCTA	ACTACAACCAGAGCGAGGMC	TACGCCAAGCTTTACTGAGCTAACTACAACCAGAGCGAGGMC
PCR2_fwd-5	TACGCCAAGCTT	CATAGTAGTG	ACTACAACCAGAGCGAGGMC	TACGCCAAGCTTCATAGTAGTGACTACAACCAGAGCGAGGMC
PCR2_fwd-6	TACGCCAAGCTT	CGAGAGATAC	ACTACAACCAGAGCGAGGMC	TACGCCAAGCTTCGAGAGATACACTACAACCAGAGCGAGGMC
PCR2_fwd-7	TACGCCAAGCTT	ATACGACGTA	ACTACAACCAGAGCGAGGMC	TACGCCAAGCTTATACGACGTAACTACAACCAGAGCGAGGMC
PCR2_fwd-8	TACGCCAAGCTT	TCACGTACTA	ACTACAACCAGAGCGAGGMC	TACGCCAAGCTTTCACGTACTAACTACAACCAGAGCGAGGMC
PCR2_rev	ACCCGGGGATCC	-	TGCCAGGTCAGTGTGATCTC	ACCCGGGGATCCTGCCAGGTCAGTGTGATCTC
